# Emergence of two novel HIV-1 Circulating Recombinant Forms (CRF190_0708 and CRF191_0708): molecular characterization and clinical insights from a five-year study in Yunnan, China

**DOI:** 10.1186/s12866-026-05323-x

**Published:** 2026-06-23

**Authors:** Li Gao, Wei Chang, Yuanlu Shu, Yang Liu, Mi Zhang, Qian Yang, Jianjian Li, HongYe Pai, Lingling Huang, Yongjiao Chen, Zhiqing Yang, Fei Lu, Xingqi Dong, Yue Feng, Xueshan Xia

**Affiliations:** 1 Faculty of Life Science and Technology & Yunnan Province Key Laboratory of Public Health and Biosafety, Kunming University of Science and Technology, Kunming, China; 2 School of Public Health, Kunming Medical University, Kunming, China; 3 Department of Infectious Diseases, Yunnan Provincial Infectious Diseases Hospital, Kunming, China; 4 Wenshan City People’s Hospital Antiviral Treatment Clinic, Wenshan, China; 5 Department of Infectious Diseases, People’s Hospital of Wenshan Zhuang and Miao Autonomous Prefecture, Wenshan, Yunnan Province China; 6 Qiu Bei County People’s Hospital Infectious Diseases Department, Wenshan, China; 7 Department of Infectious Diseases, First People’s Hospital of Xichou County, Wenshan, China; 8 Yunnan Provincial Key Laboratory of Public Health and Biosafety, Kunming Medical University, Kunming, China

**Keywords:** HIV-1, Near full-length genome, CRF, ART, Yunnan

## Abstract

**Background:**

To characterize HIV-1 molecular epidemiology and identify novel circulating recombinant forms (CRFs) among antiretroviral therapy (ART)-naïve heterosexuals in Yunnan, China, and evaluate their clinical impact.

**Methods:**

This study examined 636 HIV-1 *pol* sequences to analyze genetic diversity, pretreatment drug resistance (PDR), and transmission networks. Near full-length genomes were obtained to identify and characterize novel recombinants, with their evolutionary history inferred by Bayesian analysis. Co-receptor tropism was predicted, and the five-year clinical outcomes (including immune reconstitution and virologic response) of patients infected with the novel CRFs were compared.

**Results:**

The most prevalent type identified was CRF08_BC, accounting for 50.16% of cases. The prevalence of drug resistance was 5.97% (38/636), with the K103N mutation being the most common. An analysis of transmission networks revealed that 52.2% (272/521) of clusters were associated with CRF07_BC and CRF08_BC. Two novel second-generation CRFs were identified: CRF190_0708, with an estimated time to the most recent common ancestor (tMRCA) of 1998.9, and CRF191_0708, with a more recent tMRCA ranging from 2009.5 to 2011.6. During the five-year follow-up period, viral rebound was observed in 7 patients in the CRF190_0708 group and in 1 patient in the CRF191_0708 group. Drug-resistance mutations (M184V and K103N) were detected in a subset of rebound cases in the CRF190_0708 group.

**Conclusions:**

This study identifies two novel HIV-1 recombinants, CRF190_0708 and CRF191_0708, highlighting ongoing viral evolution in Yunnan. Preliminary findings suggest possible clinical differences, warranting further investigation. Continued molecular surveillance is needed.

**Trial registration:**

The clinical study was registered at ClinicalTrials.gov under the identifier NCT03852849. The date of registration was March 22, 2019.

**Supplementary Information:**

The online version contains supplementary material available at https://doi.org/10.1186/s12866-026-05323-x.

## Introduction

By 2024, the estimated number of new HIV infections worldwide had decreased by 40% compared with 2010 levels, with approximately 1.3 million cases reported [1]. This substantial advancement in global public health can be primarily attributed to the worldwide implementation of interventions aligned with the “90%-90%-90%” targets established by the Joint United Nations Programme on HIV/AIDS [2]. Significant advancements have been made in China, particularly in Yunnan Province, which achieved the distinction of becoming the first region in the nation to reach these objectives in 2020. The province exhibited noteworthy rates of 90.1% for diagnosis, 90.5% for treatment, and 96.3% for viral suppression [3]. However, attaining the “95%-95%-95%” targets in Yunnan poses a substantial challenge [2]. A major challenge is the province’s exceptional HIV-1 genetic diversity, which complicates diagnostic accuracy and treatment monitoring.

To address this, particular attention must be paid to the region’s specific viral landscape. In China, the genetic diversity of HIV-1 in Yunnan Province is exceptionally intricate and varied [4]. Of the 191 known circulating recombinant forms (CRFs) identified worldwide, at least 70 were first reported in China, with 29 originating from Yunnan. B/C recombinants pose a considerable and continually evolving public health challenge. Yunnan is the source of 13 out of the 21 known global CRF_BC variants, which primarily circulate among heterosexual individuals and people who inject drugs [5]. CRF07_BC primarily characterizes the local epidemic among men who have sex with men (MSM), while CRF08_BC characterizes the heterosexual population [6–8]. Furthermore, CRF85_BC, initially identified in Sichuan, is believed to have originated in Yunnan, where its prevalence has been increasing [9]. The recent discovery of second-generation recombinants (CRF160_0708 and CRF174_0708) underscores the rapid and ongoing evolution of HIV-1 in this region [10–11].

The molecular profile of HIV-1 in key regions, such as Wenshan Prefecture, a border area in Yunnan, remains poorly understood. This study conducted a molecular epidemiological analysis of newly diagnosed HIV-1 infections, focusing on viral genotype diversity, pretreatment drug resistance, and molecular transmission networks among heterosexual individuals in Wenshan Prefecture. Additionally, new CRFs of the virus were identified, and a longitudinal follow-up study was conducted. Our findings underscore the ongoing evolution of HIV-1 infection and highlight the importance of continuous surveillance and targeted interventions.

## Materials and methods

### Study participants

**Fig. 1:**
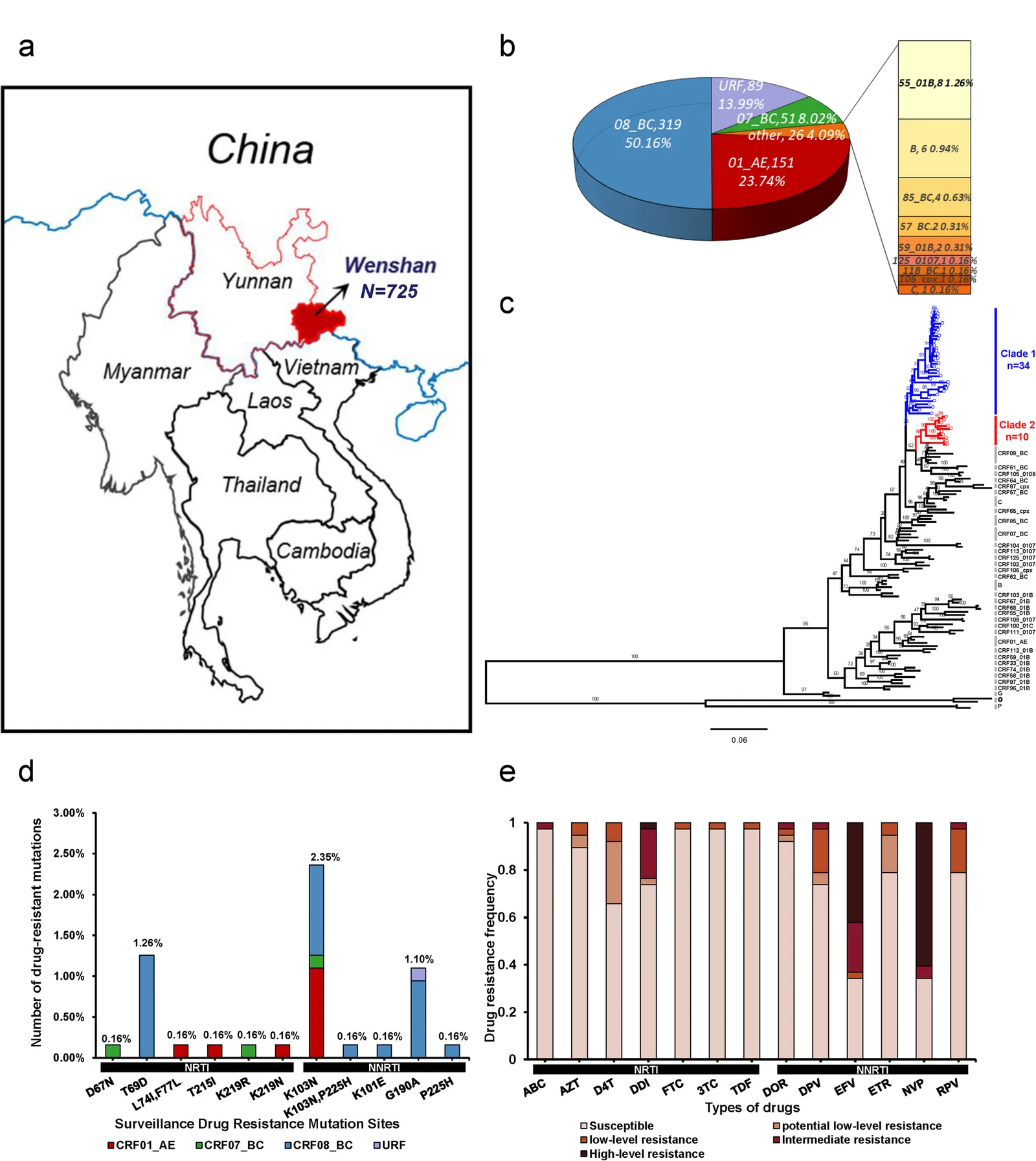
Genetic diversity and drug resistance profile of HIV-1 in Wenshan Prefecture, Yunnan Province, China. **a** Geographic location of the study area. **b** Proportional distribution of HIV-1 genotypes identified from 636 newly diagnosed individuals. **c** Maximum-likelihood phylogenetic tree of HIV-1 *pol* sequences. Two distinct clades of URFs are highlighted: Clade 1 (blue, *n* = 34) and Clade 2 (red, *n* = 10). **d** Frequencies of resistance-related mutations at different sites across various HIV-1 genotypes. **e** Resistance levels to various antiretroviral drugs. ABC, abacavir; ZDV, zidovudine; D4T, stavudine; DDI, didanosine; FTC, emtricitabine; 3TC, lamivudine; TDF, tenofovir disoproxil fumarate; DOR, doravirine; DPV, dapivirine; EFV, efavirenz; ETV, etravirine; NVP, nevirapine; RPV, rilpivirine

Between March 2019 and December 2020, we enrolled 725 ART-naïve individuals living with HIV-1 from four hospitals in Wenshan Prefecture, Yunnan Province (Wenshan People’s Hospital, *n* = 336; People’s Hospital of Wenshan Zhuang and Miao Autonomous Prefecture, *n* = 66; Qiubei County People’s Hospital, *n* = 188; Xichou County First People’s Hospital, *n* = 135) (Fig. 1a). These participants were recruited as part of a registered clinical trial (ClinicalTrials.gov identifier: NCT03852849), entitled “Cohort Study on Appropriate Strategies of the Rapid Build of HIV-Related Protective Barriers in Yunnan, China”, which aimed to evaluate intervention strategies to improve ART coverage and viral suppression among newly diagnosed HIV-infected individuals. The inclusion criteria were as follows: (1) newly diagnosed HIV-1 infection during the study period; (2) age ≥ 18 years; (3) heterosexual transmission; and (4) residence within the study area. Individuals with injection drug use or severe mental disorders were excluded. Prior to ART initiation, baseline plasma samples were collected, centrifuged, and stored at -80 °C for subsequent molecular analysis. Sociodemographic and clinical data, including baseline CD4 + T-cell counts and plasma viral loads, were extracted from medical records. All samples were de-identified to ensure confidentiality.

### Amplification and sequencing of the *pol* region and NFLG of HIV-1

HIV-1 RNA was extracted from 200 µL of serum using the Viral RNA Mini Kit (TianGen, China) according to the manufacturer’s protocol. A 1,295 bp *pol* fragment (HXB2: 2147–3441) was amplified by RT-nested PCR [12]. For near-full-length genome (NFLG) sequencing, two overlapping fragments (5.5 kb and 3.7 kb) were amplified by nested PCR using a strategy primarily based on [13]. To improve coverage of the coding region, the upstream primer design was partially adapted from [14]. All PCR products were purified and bidirectionally sequenced by TsingKe Biotech Co., Ltd. (Kunming, China). The resulting sequences were assembled using ContigExpress (v9.1). Because the 3’ terminal region showed reduced sequencing quality, low-quality nucleotides at the sequence ends were trimmed after sequence assembly. Consequently, the final sequences retained for subsequent analyses corresponded to HXB2 positions 790–9470.

### HIV-1 genotyping and drug resistance analysis

The quality of the *pol* gene sequences was verified using the online HIV-1 Quality Control tool (https://www.hiv.lanl.gov/content/sequence/QC/index.html). The initial classification of subtypes was conducted using the COMET tool (https://comet.lih.lu/index.php). After this, a phylogenetic analysis was conducted to support the genotypes. Reference sequences for the *pol* region were retrieved from the Los Alamos HIV Sequence Database and aligned using MAFFT (v7.310) with an automatic strategy. Maximum-likelihood (ML) trees were constructed in IQ-TREE (v2.4.0) under the GTR + F+R4 model, with node support assessed by 1,000 bootstrap replicates (reliable if > 90%). Trees were visualized and edited in FigTree (v1.4.4). For pretreatment drug resistance (PDR) analysis, *pol* sequences were submitted to the Stanford HIV Drug Resistance Database. Resistance to protease inhibitors (PIs), nucleoside reverse transcriptase inhibitors (NRTIs), and non-nucleoside reverse transcriptase inhibitors (NNRTIs) was interpreted using the Stanford University HIV Drug Resistance Database (https://hivdb.stanford.edu); sequences exhibiting low-, intermediate-, or high-level resistance were categorized as having PDR [15].

### Identifying the HIV-1 molecular transmission network

Pairwise genetic distances for the aligned and trimmed sequences (HXB2: 2314–3285; 972 bp) were calculated using HIV-TRACE, based on the Tamura-Nei 93 (TN93) substitution model [16]. The optimal genetic distance threshold for defining transmission clusters was identified by evaluating the range from 0.001 to 0.035 substitutions per site and selecting the value that yielded the maximum number of clusters. The resulting molecular network was then visualised using Cytoscape (v3.10.3), which consists of independent molecular clusters (≥ 2 nodes) with nodes and links.

### NFLG phylogenetic and recombination analyses

The obtained NFLG sequences were aligned with HIV-1 reference sequences, including major subtypes, CRF01_AE, and BC-related CRFs, employing the MAFFT alignment tool. An ML phylogenetic tree was reconstructed with IQ-TREE under the general time-reversible model with a gamma distribution (GTR + F+I+R7) for nucleotide substitutions. The analysis of recombination events was conducted by identifying putative breakpoints with SimPlot (v3.5.1) using bootscanning analysis. For each genomic subregion defined by these breakpoints, an ML tree was reconstructed to confirm the recombination pattern of the novel HIV-1 CRF. The genomic structures were mapped using the Recombinant HIV-1 Drawing Tool (https://www.hiv.lanl.gov/content/sequence/DRAW_CRF/recom_mapper.html).

### Bayesian MCMC evolutionary analysis

Bayesian phylogenetic analysis was performed in BEAST v1.10.5 [17] to estimate the time to the most recent common ancestor (tMRCA) of the two novel CRFs. For CRF190_0107, the four CRF08_BC‑derived regions (nt 790–2682, 3152–4569, 5035–6468, and 6646–9470) were concatenated, divided into genomic regions, and analyzed with pure CRF08_BC sequences. For CRF191_0708, the regions derived from CRF07_BC (nt 3771–5492) and CRF08_BC (nt 790–2918) were treated as continuous fragments and analysed alongside the corresponding pure sequence datasets. The temporal signal was assessed using TempEst v1.5.3. The best-fit nucleotide substitution model (GTR + I+G) was determined by jModelTest v2.1.7. We employed an uncorrelated lognormal relaxed clock and a Bayesian skyline prior. Each Markov chain Monte Carlo (MCMC) chain ran for at least 15 million generations, sampling every 1,000 steps. Convergence was confirmed in Tracer v1.7.2 (all ESS > 200). A maximum clade credibility (MCC) tree was generated from the posterior trees using TreeAnnotator (10% burn-in discarded) and visualized in FigTree.

### HIV-1 co-receptor tropism analysis

Co-receptor usage was predicted for the NFLG sequences using the Geno2Pheno[coreceptor] algorithm (https://coreceptor.geno2pheno.org), with a false-positive rate (FPR) threshold of 10% defining viral phenotypes. Sequences with an FPR ≤ 10% were classified as CXCR4-tropic or dual-tropic (R5 × 4), while those with an FPR > 10% were classified as CCR5-tropic [18].

### Statistical analysis

Statistical analyses were performed using SPSS v20.0. Group comparisons were performed via the chi-square test or Fisher’s exact test. The factors associated with clustering were analysed via binomial logistic regression. All variables were included in the multivariate model regardless of their significance in the univariate analysis. Odds ratios (ORs) and 95% confidence intervals (CIs) were calculated, with *P* < 0.05 considered statistically significant. A linear mixed-effects model (LMM) was used to assess longitudinal changes in CD4 cell count over time and to compare differences between subtypes.

## Results

### Characteristics of the study population

A total of 725 patients were enrolled, and *pol* gene sequences were successfully obtained from 636 (87.72%). Significant differences were observed in sample collection time, baseline CD4 cell count, and baseline viral load between samples with successful amplification and those with amplification failure (all *P* < 0.001) (Table S1). Consequently, all subsequent analyses were restricted to these 636 patients with complete sequence data. Among them, 68.08% (433/636) were male. The majority were aged over 30 years (92.76%, 590/636). In terms of ethnicity, 56.76% (361/636) were Han and 20.60% (131/636) were Zhuang.

### HIV-1 subtype distribution, prevalence of PDR, and molecular transmission network

Among the 636 sequences analysed, the CRF08_BC HIV-1 subtype was identified as the most prevalent, accounting for 50.16% (319/636) of the cases. Subsequent analyses identified CRF01_AE at a rate of 23.74% (151/636), followed by unique recombinant forms (URFs) at 13.99% (89/636) and CRF07_BC at 8.02% (51/636). Nine additional subtypes were identified, collectively accounting for 4.09% (26/636) of the samples (Fig. 1b). A comparative analysis of demographic characteristics across the various subtypes revealed statistically significant age differences (*P* < 0.001), educational background (*P* = 0.013), and baseline viral load (*P* < 0.001) (Table 1). Phylogenetic analysis of the *pol* gene sequences revealed two distinct monophyletic clusters that did not group with any known HIV-1 subtypes or CRFs. One cluster, designated as Clade 1, comprised 34 sequences, while the other, designated as Clade 2, comprised 10 sequences (Fig. 1c). These clusters were therefore considered potential novel recombinant lineages. The overall prevalence of PDR was 5.97% (38/636). The prevalence rates of NRTI- and NNRTI-associated mutations were 2.04% (13/636) and 3.92% (25/636), respectively. Among all subtypes, PDR was most frequently observed in CRF08_BC (7.52%, 24/319), which was marginally higher than that in the other subtypes, including CRF01_AE (6.62%, 10/151), CRF07_BC (5.88%, 3/51), and URFs (1.12%, 1/89) (Fig. 1d). The most prevalent mutation was K103N (*n* = 15), followed by T69D (*n* = 8) and G190A (*n* = 7), with single occurrences of D67N, L74I/F77L, T215I, K219R, K219N, K103N/P225H, K101E, and P225H. These mutations resulted in varying degrees of resistance to 13 antiretroviral medications (Fig. 1e).

**Table 1:** Demographic characteristics and genotypes of study subjects

Major sociodemographic variables	Total (%)	Subtype					
		CRF01_AE	CRF07_BC	CRF08_BC	Others/URFs	χ2	*P*- value^a^
Total	636(100)	151(23.74)	51(8.02)	319(50.16)	115(18.08)	246.94	**< 0.001**
Age(years)						49.094	**< 0.001**
< 30	46(7.23)	5(3.31)	11(21.57)	26(8.15)	4(3.48)		
30–39	148(23.27)	26(17.22)	19(37.25)	84(26.33)	19(16.52)		
40–49	185(29.09)	45(29.8)	14(27.45)	93(29.15)	33(28.7)		
50–59	131(20.6)	36(23.84)	2(3.92)	64(20.06)	29(25.22)		
≥ 60	126(19.81)	39(25.83)	5(9.8)	52(16.3)	30(26.09)		
Gender						3.381	0.337
Male	433(68.08)	101(66.89)	39(76.47)	210(65.83)	83(72.17)		
Female	203(31.92)	50(33.11)	12(23.53)	109(34.17)	32(27.83)		
Ethnicity						16.545	0.145
Han	361(56.76)	86(56.95)	26(50.98)	184(57.68)	65(56.52)		
Zhuang	131(20.6)	39(25.83)	6(11.76)	67(21)	19(16.52)		
Miao	70(11.01)	12(7.95)	9(17.65)	34(10.66)	15(13.04)		
Yi	64(10.06)	11(7.28)	10(19.61)	28(8.78)	15(13.04)		
Others^b^	10(1.57)	3(1.99)	0(0)	6(1.88)	1(0.87)		
Education background						24.430	**0.013**
Primary school and below	106(16.67)	32(21.19)	2(3.92)	51(15.99)	21(18.26)		
Junior middle school	45(7.08)	8(5.3)	2(3.92)	28(8.78)	7(6.09)		
High or technical secondary school	279(43.87)	55(36.42)	29(56.86)	150(47.02)	45(39.13)		
Junior college or above	9(1.42)	0(0)	0(0)	6(1.88)	3(2.61)		
Unknow	197(30.97)	56(37.09)	18(35.29)	84(26.33)	39(33.91)		
Marital status						8.046	0.530
Married	326(51.26)	79(52.32)	27(52.94)	156(48.9)	64(55.65)		
Unmarried	108(16.98)	17(11.26)	11(21.57)	62(19.44)	18(15.65)		
Divorced or widowed	165(25.94)	46(30.46)	11(21.57)	82(25.71)	26(22.61)		
Unknow	37(5.82)	9(5.96)	2(3.92)	19(5.96)	7(6.09)		
Collection Time							
2019	238(37.42)	67(44.37)	16(31.37)	111(34.8)	44(38.26)	4.884	0.180
2020	398(62.58)	84(55.63)	35(68.63)	208(65.2)	71(61.74)		
Baseline CD4 cell count (cells/mm^3^)						11.537	0.241
< 200	246(38.68)	64(42.38)	14(27.45)	123(38.56)	45(39.13)		
200–349	234(36.79)	57(37.75)	21(41.18)	109(34.17)	47(40.87)		
350–499	111(17.45)	23(15.23)	10(19.61)	65(20.38)	13(11.3)		
≥ 500	45(7.08)	7(4.64)	6(11.76)	22(6.9)	10(8.7)		
Baseline Viral load (log copies/ml)						55.854	**< 0.001**
< 3	4(0.63)	0(0)	1(1.96)	2(0.63)	1(0.87)		
3–4	52(8.18)	7(4.64)	7(13.73)	32(10.03)	6(5.22)		
4–5	318(50)	46(30.46)	30(58.82)	180(56.43)	62(53.91)		
> 5	261(41.04)	98(64.9)	13(25.49)	104(32.6)	46(40)		
Unknow	1(0.16)	0(0)	0(0)	1(0.31)	0(0)		

Values in bold indicate statistically significant differences (*P* < 0.05).

^a^ *P*-values were calculated using Pearson’s chi-square test, except for ethnicity, education level, and viral load, which were analyzed using Fisher’s exact test. ^b^ Others, the Dai, Hui, Yao, and Mongolian ethnic groups

**Fig. 2:**
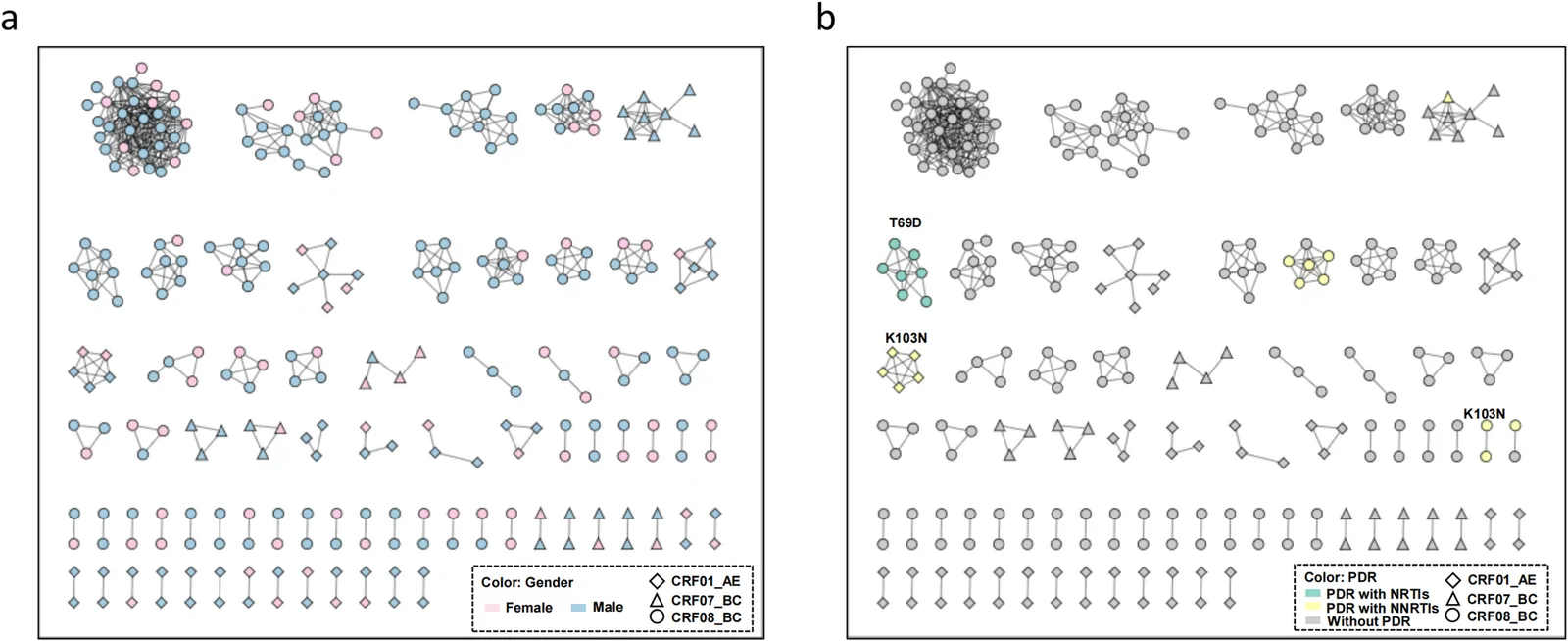
HIV transmission network in the Wenshan Prefecture. Nodes represent an individual with HIV, with different shapes indicating different genotypes. Links represent genetic relationships. **a** Colors are used to differentiate between gender groups. **b** Colors indicate the presence of PDR-related mutations

Due to limited sample sizes for minor subtypes, molecular transmission network analysis was restricted to CRF01_AE, CRF07_BC, and CRF08_BC sequences. Among the 521 sequences analysed, 272 (52.2%) were grouped into 73 transmission clusters using a genetic distance threshold of 0.017 substitutions/site. CRF08_BC formed the largest cluster, comprising 31 nodes interconnected by 188 edges. Individuals within transmission clusters were predominantly male (71.3%, 194/272) (Fig. 2a). PDR-associated mutations were identified in six transmission clusters, three of which consisted entirely of individuals carrying drug resistance mutations. Specifically, one cluster harbored NRTI mutation T69D, while two others harbored NNRTI mutation K103N (Fig. 2b). To identify factors associated with network clustering, we compared characteristics of clustered versus non-clustered individuals (Table 2). Univariate analyses identified subtype, sex, and ethnicity as risk factors for clustering. The multivariable analysis further revealed that patients with the CRF07_BC (OR: 2.593, 95% CI: 1.253–5.365) and CRF08_BC (OR: 2.873, 95% CI: 1.832–4.504) subtypes were significantly more likely to cluster than those with the CRF01_AE subtype. Females were less likely to cluster than males were (OR: 0.582, 95% CI: 0.385–0.879). In terms of ethnicity, Zhuang individuals were significantly more likely to cluster than Han individuals were (OR: 1.712, 95% CI: 1.053–2.783), whereas individuals from other ethnic groups had a lower likelihood of clustering (OR: 0.076, 95% CI: 0.008–0.687).

**Table 2:** A comparative analysis of the sociodemographic characteristics of clustered and non-clustered individuals in Wenshan Prefecture, 2019-2020

Major sociodemographic variables	No Cluster	Clusters	Univariate Analysis		Multivariable Analysis	
	No. (%)	No. (%)	OR (95% Cl)	*P*	OR (95% Cl)	*P*
Total	249 (39.15)	272 (42.76)				
Subtype						
CRF01_AE	92 (36.95)	59(21.69)	1	-	1	-
CRF07_BC	23(9.24)	28(10.29)	1.898(1.000-3.604)	**0.050**	2.593(1.253–5.365)	**0.010**
CRF08_BC	134(53.81)	185(68.01)	2.153(1.450–3.197)	**0.000**	2.873(1.832–4.504)	**0.000**
Age(years)						
18–29	22(8.84)	20(7.35)	1	-	1	-
30–39	60(24.09)	68(25)	1.265(0.630–2.541)	0.509	1.466(0.681–3.159)	0.328
40–49	77(30.92)	74(27.20)	1.044(0.527–2.068)	0.903	1.206(0.548–2.654)	0.642
50–59	47(18.88)	54(19.85)	1.237(0.603–2.541)	0.562	1.808(0.770–4.246)	0.174
≥ 60	38(15.26)	56(20.59)	1.476(0.712–3.056)	0.295	1.947(0.810–4.677)	0.136
Gender						
Male	156(62.65)	194(71.32)	1	-	1	-
Female	93(37.35)	78(28.68)	0.674(0.467–0.974)	**0.036**	0.582(0.385–0.879)	**0.010**
Ethnicity						
Han	147(59.04)	149(54.78)	1	-	1	-
Zhuang	47(18.88)	65(23.90)	1.364(0.880–2.116)	0.165	1.712(1.053–2.783)	0.030
Miao	24(9.64)	31(11.40)	1.274(0.714–2.275)	0.412	1.386(0.735–2.616)	0.313
Yi	23(9.23)	26(9.55)	1.115(0.609–2.043)	0.724	1.222(0.633–2.358)	0.550
Others	8(3.21)	1(0.36)	0.123(0.015–0.998)	**0.050**	0.076(0.008–0.687)	**0.022**
Education background						
Primary school and below	43(17.27)	42(15.44)	1	-	1	-
Junior middle school	16(6.42)	22(8.09)	1.408(0.651–3.046)	0.385	1.314(0.560–3.082)	0.531
High or technical secondary school	113(45.38)	121(44.49)	1.096(0.667–1.801)	0.717	0.971(0.561–1.681)	0.916
Junior college or above	3(1.20)	3(1.10)	1.024(0.195–5.362)	0.978	0.740(0.118–4.626)	0.748
Unknow	74(29.72)	84(30.88)	1.162(0.686–1.970)	0.577	0.933(0.516–1.689)	0.820
Marital status						
Married	122(48.99)	140(51.47)	1	-	1	-
Unmarried	49(19.68)	41(15.07)	0.729(0.451–1.179)	0.198	0.615(0.350–1.083)	0.092
Divorced or widowed	69(27.71)	70(25.73)	0.884(0.586–1.334)	0.557	0.846(0.543–1.317)	0.459
Unknow	9(3.61)	21(7.72)	2.033(0.898–4.606)	0.089	2.021(0.806–5.072)	0.134
Collection Time						
2019	89(35.45)	106(38.97)	1	-	1	-
2020	160(63.74)	166(61.03)	0.871(0.610–1.243)	0.447	0.767(0.514–1.146)	0.196
Baseline CD4 cell count (cells/mm^3^)						
< 200	98(39.36)	103(37.87)	1	-	1	-
200–349	90(36.14)	97(35.66)	1.025(0.688–1.527)	0.902	1.255(0.798–1.973)	1.255
350–499	47(18.87)	51(18.75)	1.032(0.637–1.674)	0.897	1.206(0.711–2.046)	0.486
≥ 500	14(5.62)	21(7.72)	1.427(0.687–2.963)	0.340	1.979(0.866–4.519)	0.105
Baseline Viral load (log copies/mL)						
< 3	1(0.40)	2(0.73)	1	-	1	-
3–4	30(12.05)	16(5.88)	0.267(0,022-3.171)	0.295	0.311(0.021–4.586)	0.395
4–5	116(46.59)	140(51.47)	0.603(0.054–6.739)	0.682	0.843(0.061–11.660)	0.843
> 5	101(40.56)	114(41.91)	0.564(0.050–6.317)	0.642	0.989(0.070-13.954)	0.994
Unknow	1(0.40)	0	0(0)	1.000	0(0)	1.000

*CI* confidence interval, *OR* crude odds ratio

Values in bold indicate statistically significant differences (*P *< 0.05).

### Identification of the HIV-1 CRF190_0708 and CRF191_0708

Of the 44 samples assigned to the two novel clades, only 12 yielded complete NFLG sequences: eight from Clade 1 and four from Clade 2. This was primarily due to the limited volume of samples and/or the low viral load in the remaining specimens, which made amplifying long fragments more difficult.

**Fig. 3:**
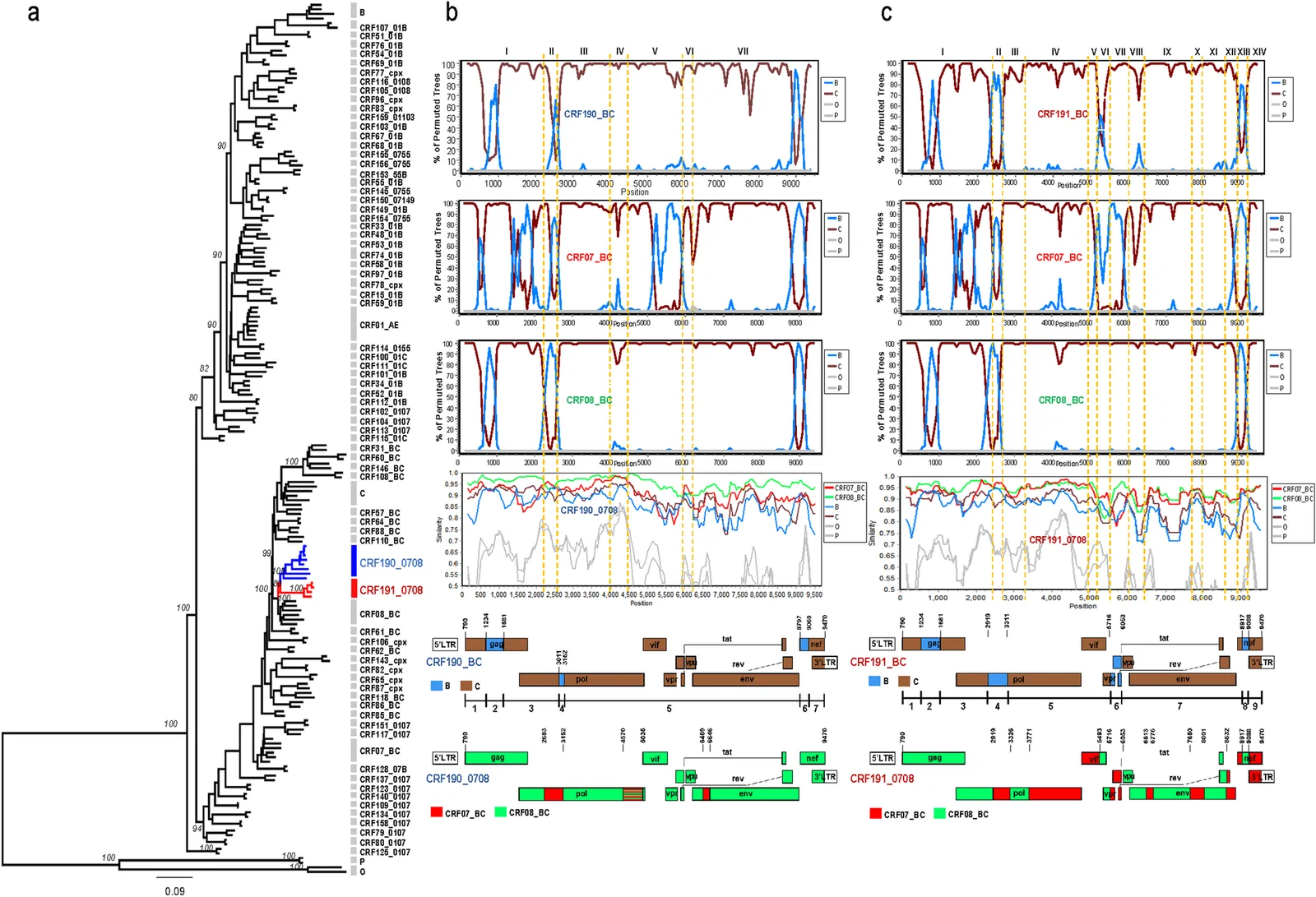
Recombination analysis of 12 NFLG sequences. **a** Maximum likelihood tree of 12 HIV-1 NFLG sequences. The blue and red branches represent CRF190_0708 and CRF191_0708, respectively. **b** Bootscan and SimPlot analyses were performed on the CRF190_0708 NFLG sequence. **c** Bootscan and SimPlot analyses were performed on the CRF191_0708 NFLG sequence. Analyses were performed with a 300-nucleotide sliding window, a 50-nucleotide step, and 1000 bootstrap replicates

The eight Clade 1 NFLG sequences formed a monophyletic cluster with 100% bootstrap support, divergent from all known HIV-1 CRFs (Fig. 3a). Bootscan analysis revealed a novel B/C recombinant with a subtype C backbone and three subtype B fragments inserted in the *gag*, *pol*, and *nef* regions (Fig. 3b). The breakpoints for fragments 1 C, 2B, 5 C, 6B, and 7 C resembled those of CRF08_BC, whereas the breakpoint for fragment 3 C resembled that of CRF07_BC [19, 20]. SimPlot analyses confirmed CRF07_BC and CRF08_BC as putative parental strains. Six breakpoints divided the genome into seven subregions (Fig. 3b). Eight NFLG sequences exhibited an identical recombinant structure and consistent breakpoint patterns (Fig. S1a). Phylogenetic trees of these subregions revealed that subregions I (nt 790–2682), III (nt 3152–4569), V (nt 5035–6468), and VII (nt 6646–9470) were associated with CRF08_BC. Subregions II (nt 2683–3151) and VI (nt 6469–6645) were associated with CRF07_BC. However, it was unclear whether subregion IV (nt 4570–5034) was derived from CRF07_BC or CRF08_BC (Fig. S2). This recombinant structure and its phylogenetic position were distinct from all previously reported CRFs. The absence of epidemiological links among the eight patients fulfilled the criteria for a novel CRF, which we provisionally designated as CRF190_0708.

The four Clade 2 sequences also formed a distinct monophyletic cluster (Fig. 3a). Bootscanning analysis revealed another B/C recombinant with a subtype C backbone and four subtype B insertions in the *gag*, *pol*, *vpr*/*tat*, and *nef* regions. The breakpoints for fragments 1 C, 2B, and 3C resembled those of CRF08_BC, whereas the breakpoints for fragments 4B, 5 C, and 6B resembled those of CRF07_BC [19, 20]. SimPlot analysis using CRF07_BC and CRF08_BC as parents revealed 13 recombination breakpoints, dividing the genome into 14 subregions (Fig. 3c). Four NFLG sequences exhibited identical recombinant structures (Fig. S1b). Phylogenetic trees of these subregions revealed that subregions I (nt 790–2918), III (nt 3326–3770), V (nt 5493–5715), VII (nt 6053–6612), IX (nt 6775–7679), XI (nt 8001–8531), and XIII (nt 8917–9087) were associated with CRF08_BC. Subregions II (nt 2919–3325), IV (nt 3771–5492), VI (nt 5716–6052), VIII (nt 6613–6774), X (nt 7680–8000), XII (nt 8532–8916), and XIV (nt 9088–9470) were associated with CRF07_BC (Fig. S3). Following the established nomenclature criteria [5], the newly identified recombinant was designated CRF191_0708. Baseline sociodemographic and clinical characteristics of the 12 individuals with novel CRFs are summarized in Table 3.

**Table 3:** Demographic characteristics of 12 HIV-1 infected individuals

Patient ID	Sampling year	Gender	Age	Ethnic group	Marriage Status	Education	CD4 cell count (cells/mm3)	Viral load (log copies/mL)	Subtype	Co-receptor tropism
W8	2019	Male	41	Han	Married	High secondary school	554	4.47	CRF190_0708	CXCR4
S57	2019	Male	57	Yi	Married	High secondary school	64	5.08	CRF190_0708	CCR5
S70	2019	Male	70	Han	Unknown	Unknown	222	4.88	CRF190_0708	CCR5
S1	2019	Male	53	Han	Unknown	Unknown	224	6.47	CRF190_0708	CCR5
W113	2019	Male	61	Han	Married	Unknown	196	5.56	CRF190_0708	CCR5
S10	2019	Male	72	Miao	Married	High secondary school	246	5.35	CRF190_0708	CCR5
S29	2019	Male	70	Han	Divorced or widowed	High secondary school	326	6.13	CRF190_0708	CCR5
W69	2019	Male	33	Han	Unmarried	High secondary school	281	4.40	CRF190_0708	CXCR4
W94	2019	Male	51	Han	Divorced or widowed	Junior middle school	195	4.82	CRF191_0708	CCR5
S40	2019	Female	45	Han	Married	High secondary school	71	5.31	CRF191_0708	CCR5
S41	2019	Male	51	Han	Married	High secondary school	420	4.50	CRF191_0708	CCR5
W56	2019	Female	35	Zhuang	Married	High secondary school	332	4.52	CRF191_0708	CCR5

### Evolutionary history of HIV-1 CRF190_0708 and CRF191_0708

**Fig. 4:**
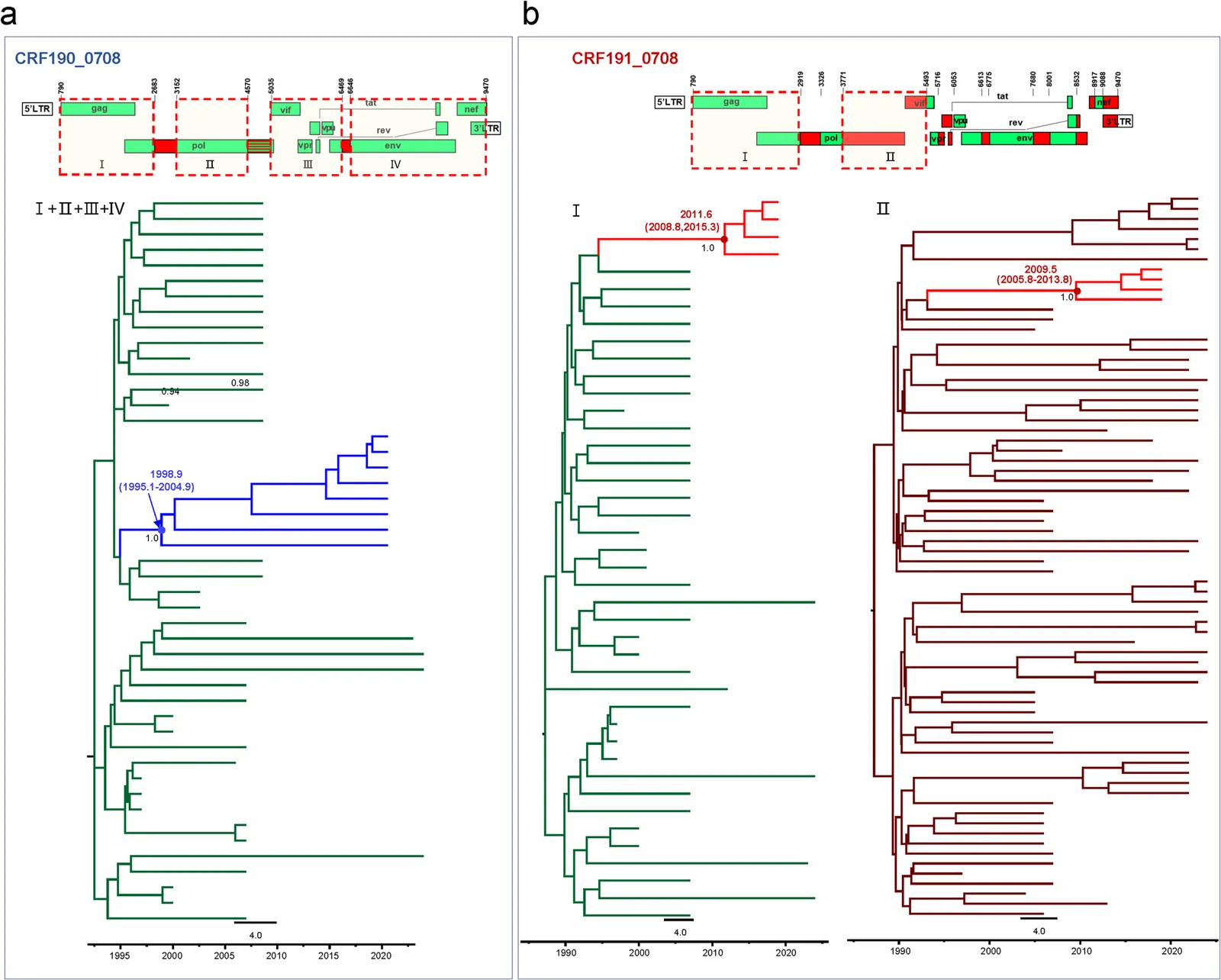
Maximum clade credibility tree of HIV-1 CRF190_0708 and CRF191_0708. **a** For CRF190_0708, one MCC tree was reconstructed using the concatenated regions derived from CRF08_BC I to IV, corresponding to nt 790–2682, 3152–4569, 5035–6468, and 6646–9470. **b** For CRF191_0708, two MCC trees were reconstructed separately using region I (derived from CRF08_BC, nt 790–2918) and region II (derived from CRF07_BC, nt 3771–5492). The blue and red solid circles represent tMRCA of CRF190_0708 and CRF191_0708, respectively. Numbers at the nodes indicate posterior probabilities, and the estimated tMRCA with 95% HPD intervals is shown for the corresponding cluster

Bayesian molecular clock analysis estimated distinct tMRCAs for the two novel CRFs. For CRF190_0708, the tMRCA was estimated at 1998.9 (95% highest posterior density [HPD]: 1995.1–2004.9). In contrast, CRF191_0708 showed a more recent origin, with tMRCA estimates of 2011.6 (95% HPD: 2008.8–2015.3) and 2009.5 (95% HPD: 2005.8–2013.8), suggesting that it likely emerged between 2009.5 and 2011.6 (Fig. 4).

### Prediction of co-receptor selection

To assess co-receptor tropism, a critical virological characteristic, the envelope gp120 V3 loop sequences from patients with CRF190_0708 and CRF191_0708 (*n* = 12) were analysed. Among the eight samples from the CRF190_0708 group, six were classified as CCR5-tropic, with an FPR range of 83.0% to 91.2%. Two samples were identified as CXCR4-tropic, with FPRs of 6.9% and 1.9%, respectively. An analysis of the CRF191_0708 group revealed that all four variants were CCR5-tropic, exhibiting an FPR range of 89.1% to 96.7%, as depicted in Table 3.

### Immune reconstitution and viral suppression in CRF190_0708 and CRF191_0708 patients during ART

**Fig. 5:**
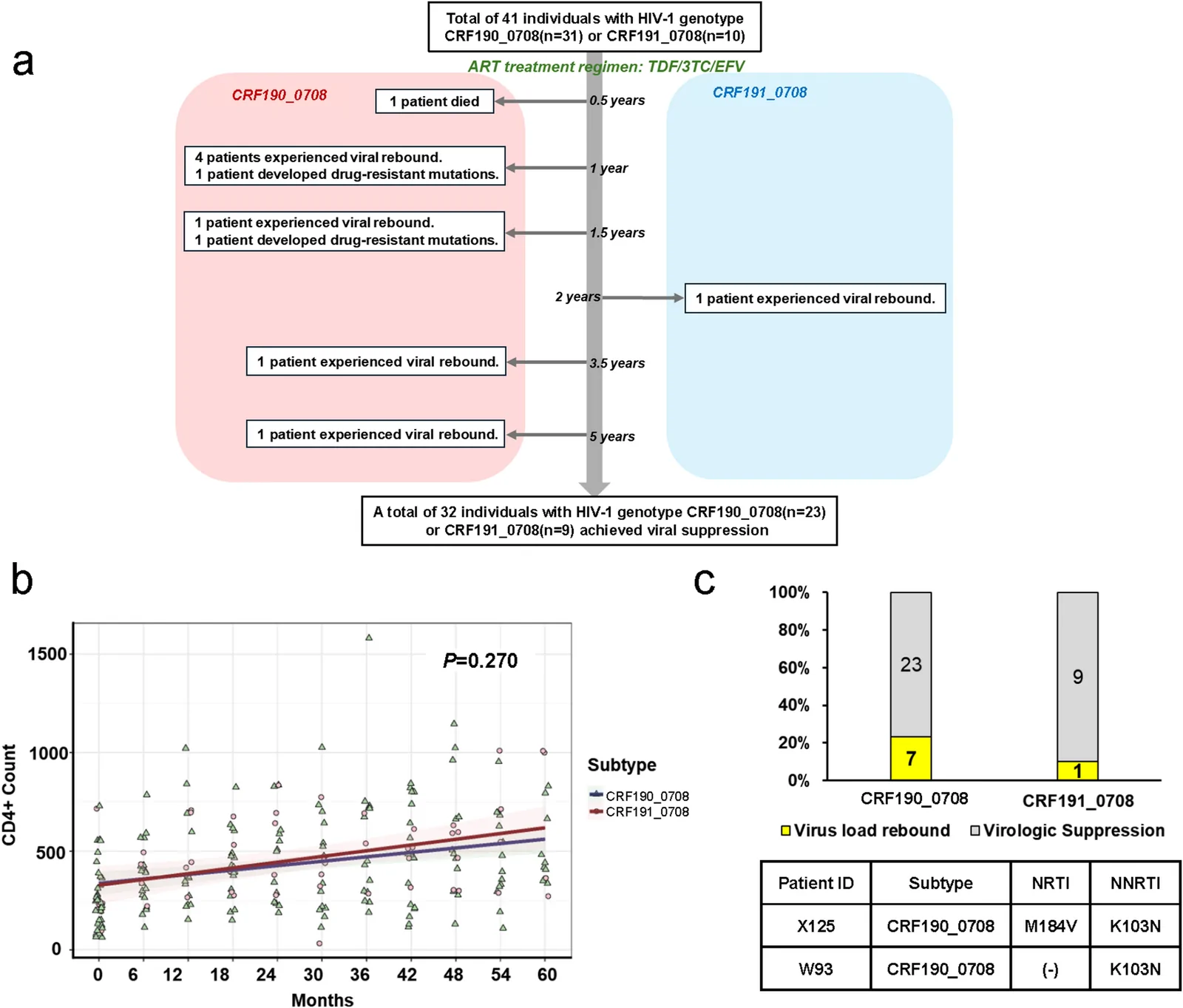
Immune Reconstitution, Viral Suppression, and Drug Resistance–Related Mutations in CRF190_0708 and CRF191_0708 Patients During ART. **a** Flowchart of patient inclusion, follow-up, and outcomes. **b** CD4⁺ T-cell count recovery over 60 months of ART. **c** Rates of virologic suppression and viral rebound. Drug resistance mutations were detected in the *pol* sequences of two CRF190_0708 patients with viral rebound

No significant differences were observed in baseline CD4 + T-cell counts or viral loads between the CRF190_0708 and CRF191_0708 groups. A five-year follow-up was conducted (Fig. 5a). Of the 34 patients with CRF190_0708, 3 were transferred to other facilities and were not included in follow-up. The remaining 31 patients were followed, among whom 1 patient died within 0.5 years. After these exclusions, 30 patients in the CRF190_0708 group and 10 patients in the CRF191_0708 group were included in the final analysis. All patients initiated first-line ART comprising predominantly tenofovir/lamivudine/efavirenz (TDF/3TC/EFV). During follow-up, regimen adjustments, including drug substitutions or dose modifications, were made in cases of treatment failure or adverse drug reactions. Viral rebound was observed in 23.3% (7/30) of patients with CRF190_0708, compared to 10.0% (1/10) in the CRF191_0708 group. Patients infected with CRF191_0708 showed a slightly improved trend toward immune reconstitution, as reflected by higher CD4 + T-cell counts following ART initiation, although this difference was not statistically significant (*P* = 0.270) (Fig. 5b) (Table S2). *Pol* sequences from two CRF190_0708 rebound samples revealed M184V and K103N resistance mutations (Fig. 5c). Overall, 32 patients achieved viral suppression, defined as a viral load consistently ≤ 200 copies/ml [21].

## Discussion

Wenshan Prefecture, a high-prevalence border region in Yunnan, now displays over 13 distinct HIV-1 genotypes, a marked increase from the 3–4 reported in 2012–2015. This heightened diversity likely stems from population mobility. The epidemic is dominated by CRF08_BC (50.16%) [22, 23], which has a history of exponential growth locally, while CRF01_AE prevalence has declined from 42.2% to 23.74%, possibly outcompeted by more transmissible strains [24, 25]. A moderate level of PDR was observed (5.97%) [26], signaling a need for enhanced ART monitoring.

Molecular transmission networks revealed larger and more densely connected clusters for CRF07_BC and CRF08_BC compared with CRF01_AE, indicating their more robust transmission chains. Zhuang ethnicity was disproportionately represented in transmission clusters, consistent with patterns observed in neighboring Guangxi Province [27]. This may reflect population demographics, dense social networks, and healthcare access disparities [28]. In addition, females were less likely to be involved in transmission clusters than males, which may reflect differences in transmission patterns between male and female populations [29]. Notably, six clusters harbored drug-resistant mutations, indicating transmission of resistant strains that may compromise long-term ART efficacy. These findings underscore the need for targeted, culturally informed interventions.

This study identified two novel HIV-1 recombinants: CRF190_0708 and CRF191_0708. Although both are second-generation recombinants derived from CRF07_BC and CRF08_BC with similar B/C recombination patterns, phylogenetic and recombination analyses revealed distinct mosaic structures. These distinct patterns underscore the increasing complexity of HIV-1 recombination in Yunnan [30]. Bayesian molecular clock analysis indicated distinct emergence times for the two CRFs. CRF190_0708 likely originated around 1998.9, whereas CRF191_0708 emerged more recently, between approximately 2009.5 and 2011.6. This difference suggests longer circulation of CRF190_0708 and a more recent recombination origin for CRF191_0708, potentially reflecting ongoing viral diversification in the region. Co-receptor tropism analysis showed that most CRF190_0708 variants and all CRF191_0708 variants were CCR5-tropic. Notably, CXCR4-tropic variants were identified in 2 of 8 CRF190_0708 samples. Given the association between CXCR4 tropism and accelerated disease progression [31], the clinical implications of this finding warrant investigation in larger cohorts.

The higher detection frequency of CRF190_0708 (*n* = 34) compared with CRF191_0708 (*n* = 10) suggests broader circulation in the studied population. The longitudinal analysis revealed differences in clinical outcomes between the two groups. Patients infected with CRF191_0708 showed an improved trend in immune reconstitution and a lower frequency of viral rebound compared to those infected with CRF190_0708. However, these differences were not statistically significant. Nevertheless, these observations are limited by small sample sizes (particularly for CRF191_0708), group imbalance, and unmeasured confounders including treatment adherence. These preliminary findings require validation in larger, appropriately powered cohort studies. These preliminary findings provide insights into the potential clinical heterogeneity of second-generation HIV-1 recombinants derived from CRF07_BC and CRF08_BC.

The increasing prevalence of HIV-1 recombinants globally poses substantial challenges for vaccine development and treatment strategies. Within China, the epidemiological landscape is influenced by both stable strains and new outbreaks. While CRF07_BC and CRF08_BC have demonstrated consistent prevalence, other CRFs, including CRF55_01B and CRF59_01B, have been associated with notable outbreaks, characterized by high transmissibility within specific networks and regions [32, 33]. These findings expand the current understanding of HIV-1 recombination in high-prevalence regions and highlight the ongoing emergence of second-generation recombinant forms. Continuous molecular surveillance and integrated epidemiological analyses will be essential for monitoring their spread and assessing their potential clinical impact.

## Conclusions

This study reports the identification and clinical characterization of two novel HIV-1 s-generation recombinants, CRF190_0708 and CRF191_0708, in Wenshan Prefecture, Yunnan. These findings contribute to understanding the evolutionary dynamics of CRF07_BC/CRF08_BC second-generation recombinants and document the expanding genetic diversity of HIV-1 in this border region. Preliminary observations suggest potential differences in clinical characteristics between these recombinants; however, further studies are required to clarify their biological and clinical significance. Continuous molecular surveillance remains essential for monitoring the emergence and spread of novel recombinant forms.

## Supplementary Information

Supplementary Material 1.

Supplementary Material 2.

Supplementary Material 3.

Supplementary Material 4.

Supplementary Material 5.

Supplementary Material 6.

## Data Availability

The data used to calculate the median values are listed in Supplementary Table S3. The DNA nucleotide sequence data supporting the findings of this study are available in GenBank. The accession numbers are as follows: PV741651-PV742286 ( *pol* region sequence), PV745739-PV745750 (NFLG sequence).

## Abbreviations

ART: Antiretroviral therapy
CIs: Confidence intervals
CRF: Circulating recombinant form
FPR: False-positive rate
HPD: Highest posterior density
LMM: Linear mixed-effects model
MCC: Maximum clade credibility
MCMC: Markov chain Monte Carlo
ML: Maximum-likelihood
MSM: Men who have sex with men
NFLG: Near-full-length genome
NNRTIs: Non-nucleoside reverse transcriptase inhibitors
NRTIs: Nucleoside reverse transcriptase inhibitors
ORs: Odds ratios
PDR: Pretreatment drug resistance
PIs: Protease inhibitors
TDF/3TC/EFV: Tenofovir/lamivudine/efavirenz
tMRCA: Time to the most recent common ancestor
TN93: Tamura-Nei 93
URF: Unique recombinant form
