# Supplementary material for: Emergence of two novel HIV-1 Circulating Recombinant Forms (CRF190_0708 and CRF191_0708): molecular characterization and clinical insights from a five-year study in Yunnan, China
Source: BMC Microbiol. 2026 Jun 23;26:774. doi: 10.1186/s12866-026-05323-x (PMC13540875; doi:10.1186/s12866-026-05323-x)
Supplement: Supplementary file 1 [file 12866_2026_5323_MOESM1_ESM.docx]

**Table S1.** Sociodemographic characteristics of newly diagnosed HIV-infected patients and those with successfully obtained pol sequences in Wenshan from 2019 to 2020.

| Major sociodemographic variables | | | Total n=725  (%) | *pol* region sequences obtained n=636  (%) | | χ2 | *P-value* ^a^ |  |
| --- | --- | --- | --- | --- | --- | --- | --- | --- |
| Age(years) | |  | |  | | 2.467 | 0.651 |  |
|  | <30 | | 56(7.72) | 46(7.23) |  | | |  |
|  | 30–39 | | 168(23.17) | 148(23.27) |  | | |  |
|  | 40–49 | | 208(28.69) | 185(29.09) |  | | |  |
|  | 50–59 | | 147(20.28) | 131(20.6) |  | | |  |
|  | ≥ 60 | | 146(20.14) | 126(19.81) |  | | |  |
| Gender | |  | |  | | 0.115 | 0.735 |  |
|  | Male | | 492(67.86) | 433(68.08) |  | | |  |
|  | Female | | 233(32.14) | 203(31.92) |  | | |  |
| Ethnicity | |  | |  | | 4.753 | 0.314 |  |
|  | Han | | 419(57.79) | 361(56.76) |  | | |  |
|  | Zhuang | | 143(19.72) | 131(20.6) |  | | |  |
|  | Miao | | 78(10.76) | 70(11.01) |  | | |  |
|  | Yi | | 72(9.93) | 64(10.06) |  | | |  |
|  | Others ^b^ | | 13(1.79) | 10(1.57) |  | | |  |
| Collection Time | |  | |  | | 57.269 | **<0.001** |  |
|  | 2019 | | 309(42.62) | 238(37.42) |  | | |  |
|  | 2020 | | 416(57.38) | 398(62.58) |  | | |  |
| Baseline CD4 cell count (cells/mm^3^) | | | |  | | 224.767 | **<0.001** |  |
|  | <200 | | 249(34.34) | 246(38.68) |  | | |  |
|  | 200-349 | | 234(32.28) | 234(36.79) |  | | |  |
|  | 350-499 | | 138(19.03) | 111(17.45) |  | | |  |
|  | ≥500 | | 102(14.07) | 45(7.08) |  | | |  |
|  | Unknow | | 2(0.28) | 0 |  | | |  |
| Baseline Viral load (log copies/mL) | | | |  | 551.986 | | **<0.001** |  |
|  | <3 | | 16(2.20) | 4(0.63) |  | | |  |
|  | 3-4 | | 53(7.31) | 52(8.18) |  | | |  |
|  | 4-5 | | 325(44.83) | 318(50) |  | | |  |
|  | >5 | | 268(36.96) | 261(41.04) |  | | |  |
|  | Unknow | | 63(8.69) | 1(0.16) |  | | |  |

^a^ *P-values* were calculated using Pearson’s chi-square test, except for CD4 count, which was analyzed using Fisher’s exact test. ^b^ Others, the Dai, Hui, Yao, and Mongolian ethnic groups.
