# Supplementary material for: Emergence of two novel HIV-1 Circulating Recombinant Forms (CRF190_0708 and CRF191_0708): molecular characterization and clinical insights from a five-year study in Yunnan, China
Source: BMC Microbiol. 2026 Jun 23;26:774. doi: 10.1186/s12866-026-05323-x (PMC13540875; doi:10.1186/s12866-026-05323-x)
Supplement: Supplementary file 2 [file 12866_2026_5323_MOESM2_ESM.docx]

**Table S2.** Baseline CD4 cell counts and viral loads in patients with the CRF190_0708 and CRF191_0708 genotypes, and CD4 cell count values in patients followed for five years of treatment.

| Patient ID | CD4 cell count (cells/mm3) | | | | | | | | | | | Baseline Viral load (log copies/mL) | Subtype |
| --- | --- | --- | --- | --- | --- | --- | --- | --- | --- | --- | --- | --- | --- |
|  | Baseline | 0.5 years | 1 year | 1.5 years | 2 years | 2.5 years | 3 years | 3.5 years | 4 years | 4.5 years | 5 years |  |  |
| S57 | 64 | 114 | - | 150 | - | 114 | - | 115 | - | 109 | - | 5.08 | CRF190_0708 |
| X320 | 65 | - | - | 202 | - | 202 | - | 129 | - | 321 | - | 4.85 | CRF190_0708 |
| S40 | 71 | 222 | - | 293 | - | 383 | - | 514 | - | 548 | - | 5.31 | CRF191_0708 |
| S90 | 82 | 179 | - | 191 | - | 168 | - | 213 | - | 192 | - | 5.15 | CRF190_0708 |
| X210 | 96 | - | 266 | - | 380 | - | 285 | - | 300 | - | 272 | 4.43 | CRF191_0708 |
| W93 | 98 | - | 154 | - | 188 | - | 190 | - | 131 | - | 356 | 5.36 | CRF190_0708 |
| X213 | 110 | - | 220 | - | 234 | - | 242 | - | - | - | - | 5.28 | CRF190_0708 |
| X404^3^ | 124 | 277 | 364 | - | 508 | - | 1580 | - | 295 | - | 408 | 5.97 | CRF190_0708 |
| X88 | 133 | 204 | 228 | 490 | 288 | - | 292 | - | 412 | - | 419 | 4.57 | CRF190_0708 |
| X108 | 151 | - | 228 | - | 224 | - | 298 | - | 277 | - | 350 | 5.58 | CRF190_0708 |
| S50 | 151 | 214 | - | 272 | - | 219 | - | 207 | - | 295 | - | 3.86 | CRF190_0708 |
| W94 | 195 | - | 445 | - | 694 | - | 693 | - | 631 | - | 1009 | 4.82 | CRF191_0708 |
| W113 | 196 | 391 | - | 467 | - | 541 | - | 800 | 507 | - | 795 | 5.56 | CRF190_0708 |
| X294^3^ | 198 | 265 | - | - | 343 | - | 247 | 168 | - | - | - | 4.92 | CRF190_0708 |
| W68 | 204 | 338 | - | 277 | - | 323 | - | - | 466 | 289 | - | 4.29 | CRF191_0708 |
| I15^3^ | 214 | 299 | - | 306 | - | 296 | - | 229 | - | - | - | 4.48 | CRF190_0708 |
| S1 | 224 | - | 334 | 395 | - | 735 | - | 642 | - | 396 | - | 6.47 | CRF190_0708 |
| X249 | 231 | - | 417 | - | 642 | - | 539 | - | 597 | - | - | 4.87 | CRF191_0708 |
| X19^3^ | 236 | - | 379 | - | 277 | 32 | - | 316 | 304 | - | 364 | 4.44 | CRF191_0708 |
| I57 | 239 | - | 274 | 377 | - | 337 | 352 | - | - | 332 | - | 4.70 | CRF190_0708 |
| S10 | 246 | 399 | - | 393 | - | 211 | - | 465 | - | 357 | - | 5.35 | CRF190_0708 |
| X101 | 252 | - | - | 390 | 241 | - | - | 337 | 464 | - | 337 | 4.56 | CRF190_0708 |
| X248^3^ | 262 | - | 697 | 676 | - | 440 | - | - | - | - | - | 4.96 | CRF191_0708 |
| I21 | 268 | 310 | - | 420 | - | 476 | - | 487 | - | - | - | 4.47 | CRF190_0708 |
| X379 | 273 | 424 | - | 617 | - | - | 674 | 843 | - | 954 | - | 4.50 | CRF190_0708 |
| W69 | 281 | - | 692 | - | 588 | - | 730 | 822 | - | 683 | - | 4.40 | CRF190_0708 |
| I69 | 312 | 399 | - | 475 | - | 701 | 450 | - | - | - | - | 4.78 | CRF190_0708 |
| S29 | 326 | 568 | - | 604 | - | - | 719 | 739 | - | 697 | - | 6.13 | CRF190_0708 |
| W56 | 332 | 494 | - | 399 | - | 774 | - | 520 | - | 1010 | - | 4.52 | CRF191_0708 |
| X50 | 364 | - | 841 | - | 829 | - | - | 802 | 962 | - | 665 | 6.26 | CRF190_0708 |
| X125^3^ | 371 | - | 371 | - | 500 | - | - | 466 | - | 627 | 442 | 4.49 | CRF190_0708 |
| X262^3^ | 389 | - | 1022 | - | 836 | - | 717 | - | 663 | - | - | 5.22 | CRF190_0708 |
| S41^3^ | 420 | 434 | - | 532 | - | 456 | - | 464 | - | 713 | - | 4.50 | CRF191_0708 |
| Q29^3^ | 446 | 429 | 348 | - | 551 | - | 430 | - | 486 | - | 482 | 4.85 | CRF190_0708 |
| S61^3^ | 494 | 569 | - | 635 | - | 643 | - | 567 | - | 546 | - | 4.39 | CRF190_0708 |
| X277 | 554 | 786 | - | 824 | - | 1027 | - | - | 1024 | - | - | 4.61 | CRF190_0708 |
| W8 | 554 | - | 598 | - | 608 | - | 753 | - | 673 | - | 830 | 4.47 | CRF190_0708 |
| I18 | 558 | 337 | - | 428 | - | 524 | - | 481 | - | 478 | - | 3.77 | CRF190_0708 |
| W87 | 716 | - | 708 | - | 835 | - | - | 613 | 592 | - | 1000 | 4.82 | CRF191_0708 |
| X341 | 729 | 592 | - | - | - | - | - | - | 1146 | - | - | 4.80 | CRF190_0708 |
| X79^1^ | 88 | - | - | - | - | - | - | - | - | - | - | 5.54 | CRF190_0708 |
| S67^1^ | 206 | - | - | - | - | - | - | - | - | - | - | 5.87 | CRF190_0708 |
| S70^1^ | 222 | - | - | - | - | - | - | - | - | - | - | 4.88 | CRF190_0708 |
| X180^2^ | 198 | - | - | - | - | - | - | - | - | - | - | 5.14 | CRF190_0708 |

^1^: Patients X79, S67, and S70 were transferred to different treatment centres and therefore did not participate in the follow-up study.

^2^: Patient X180 died during treatment.

^3^: Patients underwent ART regimen modifications during follow-up due to treatment failure or drug-related adverse effects.
