# Supplementary material for: Emergence of two novel HIV-1 Circulating Recombinant Forms (CRF190_0708 and CRF191_0708): molecular characterization and clinical insights from a five-year study in Yunnan, China
Source: BMC Microbiol. 2026 Jun 23;26:774. doi: 10.1186/s12866-026-05323-x (PMC13540875; doi:10.1186/s12866-026-05323-x)
Supplement: Supplementary file 4 [file 12866_2026_5323_MOESM4_ESM.docx]

**Fig S2.** Subregional ML tree analysis of CRF190_0708. The CRF07_BC and CRF08_BC subtype clusters across the seven ML trees are highlighted with red and green backgrounds, respectively. Red branches represent the samples from this study, while black branches correspond to reference sequences.
