# Supplementary material for: Emergence of two novel HIV-1 Circulating Recombinant Forms (CRF190_0708 and CRF191_0708): molecular characterization and clinical insights from a five-year study in Yunnan, China
Source: BMC Microbiol. 2026 Jun 23;26:774. doi: 10.1186/s12866-026-05323-x (PMC13540875; doi:10.1186/s12866-026-05323-x)
Supplement: Supplementary file 6 [file 12866_2026_5323_MOESM6_ESM.docx]

| **Section / Topic** | **No** | **CONSORT checklist item description** | **Reported on page no.** |
| --- | --- | --- | --- |
| **Title and abstract** | | |  |
| Title and structured abstract | 1a | Identification as a randomised trial | NA |
|  | 1b | Structured summary of the trial design, methods, results, and conclusions | 2 |
| **Open science** | | |  |
| Trial registration | 2 | Name of trial registry, identifying number | 2 |
| Protocol and statistical analysis plan | 3 | Where the trial protocol and statistical analysis plan can be accessed | Clinical trial protocol available through ClinicalTrials.gov (NCT03852849). |
| Data availability | 4 | DNA nucleotide sequence data are available in GenBank, and supporting data are provided in Supplementary Table S2. | 13 |
| Funding and conflicts of interest | 5a | Sources of funding, and role of funders in the design, conduct, analysis and reporting of the trial | 13 |
|  | 5b | Financial and other conflicts of interest of the manuscript authors | 12,13 |
| **Introduction** | | |  |
| Background and rationale | 6 | Scientific background and rationale | 3 |
| Objectives | 7 | Specific objectives related to benefits and harms | 3 |
| **Methods** | | | **4** |
| Study participants | 8 | Details of patient or public involvement in the design, conduct and reporting of the trial | NA |
| Trial design | 9 | Description of trial design including type of trial (e.g., parallel group, crossover), allocation ratio, and framework (e.g., superiority, equivalence, non-inferiority, exploratory) | NA |
| Outcomes | 10 | Completely defined pre-specified primary and secondary outcome measures,including how and when they were assessed | 6-9 |
| Sample size | 11a | How sample size was determined, including all assumptions supporting the sample size calculation | NA |
|  | 11b | Explanation of any interim analyses and stopping guidelines | NA |
| Randomisation: |  |  | NA |
| Sequence generation | 12a | Who generated the random allocation sequence and the method used | NA |
|  | 12b | Type of randomisation and details of any restriction (e.g., stratification, blocking and block size) | NA |
| Implementation | 13 | Whether the personnel who enrolled and those who assigned participants to the interventions had access to the random allocation sequence | NA |
| Statistical methods | 14a | Statistical methods used to compare groups for primary and secondary outcomes, including harms | 6 |
|  | 14b | Definition of who is included in each analysis (e.g., all randomised participants), and in which group | 6, 9 |
|  | 14c | How missing data were handled in the analysis | 6, 9 |
|  | 14d | Methods for any additional analyses (e.g., subgroup and sensitivity analyses), distinguishing prespecified from post-hoc | 6 |
| **Results** | | |  |
| Characteristics of the study population | 15 | Clinical sample description | 6 |
| Baseline information | 16 | HIV-1 subtype distribution,prevalence of PDR,and molecular transmission network | 6, 7 |
| New CRF identification | 17 | Identification of the HIV-1 CRF190_0708 and CRF191_0708 | 8 |
| New CRF evolutionary history | 18 | Evolutionary history of HIV-1 CRF190_0708 and CRF191_0708 | 9 |
| Prediction of co-receptor tropism | 19 | Assess co-receptor tropism, a critical virological characteristic, the envelope gp120 V3 loop sequences from patients with CRF190_0708 and CRF191_0708 | 9 |
| Therapeutic response | 20 | Immune Reconstitution and Viral Suppression in CRF190_0708 and CRF191_0708 Patients During ART | 9 |
| **Discussion/Conclusions** | | |  |
| Interpretation | 21 | Interpretation consistent with results, balancing benefits and harms, and considering other relevant evidence | 10,11 |
| Limitations | 22 | Trial limitations, addressing sources of potential bias, imprecision, generalisability, and, if relevant, multiplicity of analyses | 11 |
