# Supplementary material for: Emergence of two novel HIV-1 Circulating Recombinant Forms (CRF190_0708 and CRF191_0708): molecular characterization and clinical insights from a five-year study in Yunnan, China
Source: BMC Microbiol. 2026 Jun 23;26:774. doi: 10.1186/s12866-026-05323-x (PMC13540875; doi:10.1186/s12866-026-05323-x)
Supplement: Supplementary file 7 [file 12866_2026_5323_MOESM3_ESM.docx]

**Fig S1.** SimPlot analysis of NFLG sequences. (a) CRF190_0708. (b) CRF191_0708.
